# Effects of Trehalose on Halitosis: A Randomized Cross-Over Clinical Trial

**DOI:** 10.3390/healthcare13060619

**Published:** 2025-03-13

**Authors:** Hisataka Miyai, Takaaki Tomofuji, Hirofumi Mizuno, Manabu Morita, Momoko Nakahara, Kota Kataoka, Ichiro Sumita, Yurika Uchida, Naoki Toyama, Aya Yokoi, Reiko Yamanaka-Kohno, Noriko Takeuchi, Takayuki Maruyama, Daisuke Ekuni

**Affiliations:** 1 Department of Preventive Dentistry, Faculty of Medicine, Dentistry and Pharmaceutical Sciences, Okayama University, Okayama 700-8558, Japan or shiotsudent@gmail.com (H.M.); mizuno.h19860219@gmail.com (H.M.); pu171qxi@s.okayama-u.ac.jp (N.T.); yokoi-a1@cc.okayama-u.ac.jp (A.Y.); t-maru@md.okayama-u.ac.jp (T.M.); 2 Shiotsu Dental Clinic, Kurashiki 710-0142, Japan; 3 Department of Community Oral Health, School of Dentistry, Asahi University, Mizuho 501-0296, Japan; tomofu@dent.asahi-u.ac.jp; 4 Chiaki Ajisai Dental Clinic, Ichinomiya 491-0812, Japan; 5 Department of Oral Health Sciences, Faculty of Health Care Sciences, Takarazuka University of Medical and Health Care, Osaka 531-0071, Japan; m.morita@tumh.ac.jp; 6 Department of Preventive Dentistry, Division of Dentistry, Okayama University Hospital, Okayama 700-8558, Japan; pric37ll@s.okayama-u.ac.jp (M.N.); de18017@s.okayama-u.ac.jp (K.K.); pj0p7nno@s.okayama-u.ac.jp (I.S.); y-uchida@okayama-u.ac.jp (Y.U.); reiko_y@md.okayama-u.ac.jp (R.Y.-K.); takeuti@md.okayama-u.ac.jp (N.T.); 7 Advanced Research Center for Oral and Craniofacial Sciences, Okayama University Dental School, Okayama 700-8558, Japan

**Keywords:** halitosis, trehalose, oral dryness, cross-over study, randomized trial

## Abstract

**Background/Objectives:** Halitosis is a condition characterized by an unpleasant malodor. Intra-oral halitosis is caused by volatile sulfur compounds (VSCs) and can be associated with oral dryness. Trehalose is one of the materials used to relieve oral dryness. The aim of the present study was to investigate the effect of trehalose on halitosis. **Methods:** This prospective, double-blinded, placebo-controlled, cross-over study enrolled volunteers from Okayama University Hospital. The participants were randomly divided into two groups, with one group receiving trehalose (a 10% trehalose solution) and the other receiving a placebo (distilled water) in a 1:1 allocation. The primary study outcome was the subjective organoleptic test. The secondary outcomes were the concentrations of the VSCs, which were measured using a portable gas chromatography device, and the oral moisture status, which was measured using an oral moisture meter. The planned sample size was 10 participants based on the previous study. **Results:** The final intention-to-treat analysis was performed using the data from 9 participants. After applying 10% trehalose as an oral spray, the organoleptic score decreased in a time-dependent manner. However, no significant differences were seen between the trehalose and placebo groups. In terms of secondary outcomes, the oral moisture levels increased immediately after the trehalose spray application, and significant differences in the amount of change from the baseline were seen between the trehalose and placebo groups (*p* = 0.047). No significant differences were seen in any of the other variables (*p* > 0.05). **Conclusions:** We could not identify any positive effects on halitosis from a one-time 10% trehalose application as an oral spray in this prospective, double-blinded, placebo-controlled, cross-over study. However, the trehalose application immediately improved the oral moisture levels and was useful for treating oral dryness.

## 1. Introduction

Halitosis is a condition characterized by an unpleasant malodor [1]. One review suggested a pooled prevalence for halitosis of 0.32 (95% confidence interval 0.25–0.39) [2]. Halitosis can cause embarrassment and lead to the avoidance of social contact [3].

Halitosis is divided into physiologic and pathological halitosis [1]. Pathological halitosis is subdivided into intra-oral or extra-oral pathological halitosis [4]. In intra-oral halitosis, many microbial pathogens, including gram-negative anaerobes, have been identified as producing malodorous chemical compounds [1,5]. The unpleasant malodor may be generated by the complex interplay of diverse oral microbiomes [6]. The etiologies of extra-oral halitosis may originate in the nasopharynx, lungs, or gastrointestinal tract [7]. Several extra-oral factors have been identified, including viral hepatitis B, adenoid hypertrophy, small intestinal bacterial overgrowth, and *Helicobacter pylori* infection [1,8,9]. Most cases of halitosis originate in the oral cavity, representing intra-oral halitosis or ‘oral malodor’ [10,11,12]. Intra-oral halitosis is typically caused by volatile sulfur compounds (VSCs) such as methyl mercaptan, hydrogen sulfide, and dimethyl sulfide [13]. Intra-oral halitosis can be associated with the presence of tongue coating, inflammation, carious lesions, overhanging restorations, and oral dryness [14,15].

Dryness of the oral cavity may contribute to various signs and symptoms, such as burning sensations, tongue pain, and difficulty swallowing [16]. Oral care agents such as trehalose have been applied to attenuate such problems [16,17,18,19]. Trehalose is a non-reducing disaccharide composed of two glucose molecules linked by an α,α-1,1 bond [16], and it has been shown to improve desiccation tolerance by preserving the structure and function of biological macromolecules [20]. Trehalose is considered a potential therapeutic agent for various pathologies, including glucose homeostasis [21], corneal epithelial healing [22], and dryness of the oral cavity by protecting the cell membrane [16]. As oral dryness affects halitosis, the improvement of oral dryness through the use of trehalose may have the potential for halitosis treatment. However, its efficacy against halitosis has not been clarified.

We hypothesized that trehalose may attenuate oral dryness and contribute to the improvement of halitosis. The present study, therefore, aimed to investigate the effects of trehalose on halitosis in a prospective, double-blinded, placebo-controlled, cross-over study.

## 2. Materials and Methods

### 2.1. Trial Design

The trial was a prospective, double-blind, placebo-controlled, cross-over study that enrolled participants from Okayama University Hospital in Japan. The study was conducted in accordance with the Declaration of Helsinki and the Consolidated Standards of Reporting Trials (CONSORT) guidelines. Two starting groups were set (the trehalose and placebo groups, as defined below) with an allocation ratio of 1:1. The study protocol was approved by the ethics committee at Okayama University Graduate School of Medicine, Dentistry and Pharmaceutical Sciences and Okayama University Hospital (approval no. d11005). All of the participants provided written informed consent prior to their enrollment. An independent dentist (M.M.) reviewed the safety data during the study period. The code breaking was performed after the final statistical analysis. We registered the study with the University Hospital Medical Information Network Center (UMIN) clinical trial registration system (UMIN000056596). There were no changes in the eligibility criteria or outcomes throughout the study.

### 2.2. Participants

The volunteers were recruited between December 2014 and March 2016 from Okayama University Hospital. The inclusion criteria were selected to maximize the chance that a participant would complete the trial, regardless of whether the participant reported awareness about halitosis. Participants of any sex were eligible if they met the following criteria: ≥20 years old; reported xerostomia (a subjective perception of dry mouth associated with salivary gland hypofunction) [23]; and voluntarily agreed to participate in the research after providing written informed consent. The exclusion criteria comprised the following: severe dental caries and/or periodontitis; pregnancy; allergy to foods and drugs; or a status otherwise judged by an investigator as inappropriate for the trial.

**Figure 1:**
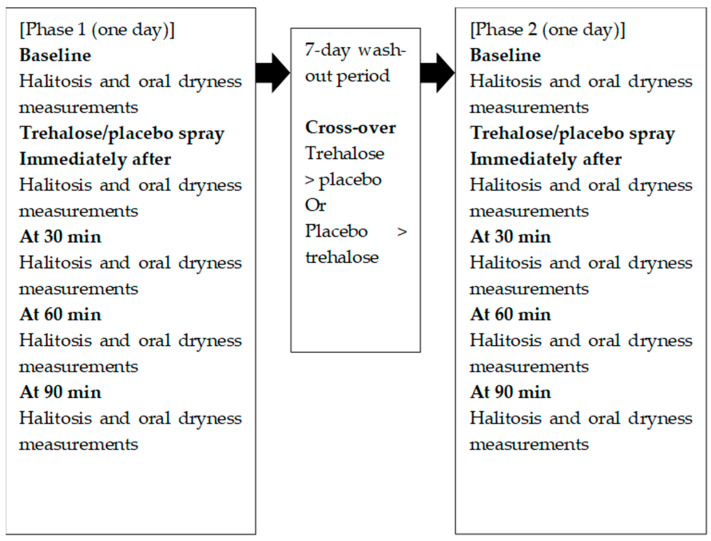
Study design.

The participants were randomly assigned by a dentist (T.T.) to either the placebo or trehalose group for phase 1, after which they completed a 7-day wash-out period followed by phase 2 (Figure 1). In phases 1 and 2, the participants received five measurements (at baseline, immediately after spray, and at 30 min, 60 min, and 90 min) (Figure 1). The participants attended hospital visits at the beginning and end of both phase 1 and phase 2, and they received 6000 JPY (approximately 37 EUR) at the end of the study after completing all of the study activities.

### 2.3. Intervention

We used trehalose (Hayashibara Biochemical Laboratories, Okayama, Japan) and distilled water (Otsuka Pharmaceutical Factory, Tokushima, Japan). In the trehalose group, we used an oral spray (0.4 mL of a 10% trehalose solution) that was applied on the dorsum of the tongue to investigate the clinical efficacy and acceptability of trehalose [16]. In the placebo group, an oral spray of distilled water (0.4 mL) was applied.

### 2.4. Outcome Assessment

The primary study outcome was the subjective organoleptic test, which serves as the gold standard for the diagnosis and evaluation of halitosis [24]. The organoleptic test result is recorded as a score from 0 (no appreciable odor) to 5 (extremely strong malodor). When the organoleptic score was >1, a dentist (H.M. (Hisataka Miyai)) diagnosed halitosis. A blinded examiner (H.M. (Hisataka Miyai)) performed the test between 08:00 and 11:00.

The secondary outcomes were the concentrations of VSCs and the oral moisture status. The same blinded examiner (H.M. (Hisataka Miyai)) performed these measurements.

The examiner used a portable gas chromatography device (Oral Chroma; Nissha FIS, Osaka, Japan). The device measures the concentrations of three VSCs (hydrogen sulfide, methyl mercaptan, and dimethyl sulfide). The examiner asked the participants not to consume garlic, onions, or alcohol or to use antiseptic mouthwash in the 24 h prior to the measurements. On the same day as the measurement, we also asked them not to eat breakfast, brush their teeth, smoke, or use perfume. Furthermore, we asked them to drink water or gargle 1 h prior to the measurements, if needed. The examiner collected air from the oral cavity following the instructions from the manufacturer. The examiner then asked the participants to keep their mouths closed for 30 s. Next, the examiner inserted a syringe into the mouth of each participant to collect 1 mL of air. The examiner injected the sample into the device using a needle [25].

The oral moisture level was measured by two dentists (H.M. (Hirofumi Mizuno) and H.M. (Hisataka Miyai)) using an oral moisture meter (Mucus; Life Co., Saitama, Japan) placed at the center of the tongue [26], and the average of two measurements was taken as the final value [27]. A lower value indicated a lower oral moisture level.

To check the intra- and inter-examiner agreement, measurements of the oral moisture level were recorded and repeated within a 2-week interval in two randomly selected volunteers. The data were analyzed with the non-parametric κ test, and the intra-class correlation was determined. The κ coefficients for the intra- and inter-examiner and intra-class correlation coefficients were >0.8.

Two dentists ((H.M. (Hirofumi Mizuno) and H.M. (Hisataka Miyai)) examined whether severe dental caries and/or severe periodontitis were present in each participant by inspection and palpation. The participants’ oral conditions were also investigated during the trials.

### 2.5. Adverse Events and Safety Monitoring

Systemic conditions and oral symptoms were recorded during the trials. A dentist (M.M.) investigated the presence of adverse events and performed safety monitoring.

### 2.6. Sample Size Calculation

The sample size was estimated assuming a difference in the organoleptic scores of 1.5 between the trehalose and placebo groups, based on a previous report [28]. A sample size of 8 participants (4 in each group in phase 1) was determined to be necessary to provide 80% power with an alpha of 0.05 for two-tailed and unpaired t tests using Sample Power (IBM, Tokyo, Japan). Assuming an attrition rate of 10%, the planned sample size was 10 participants.

### 2.7. Randomization

Each selected participant received a code number, and a study coordinator (T.T.) used a computer-generated table to randomly allocate patients to one of the two groups (trehalose or placebo group at phase 1) using an allocation ratio of 1:1. The coordinators kept the sequentially numbered list in a sealed envelope.

### 2.8. Blinding

The study personnel, including the examiners and the investigator responsible for the data analysis, were blinded to the treatment assignments. All of the participants were likewise blinded. Only the coordinator (T.T.) knew the treatment assignments.

### 2.9. Statistical Analysis

The data analysis was performed using SPSS version 25 (IBM). The data are summarized as the mean ± standard deviation for the continuous variables and the frequency and percentage for the categorical variables. We used the Mann–Whitney *U* test for the analysis of the primary outcome and the intention-to-treat (ITT) analysis for the secondary outcomes. A significance level of 0.05 was applied for all of the statistical analyses.

## 3. Results

### 3.1. Flow Chart

**Figure 2:**
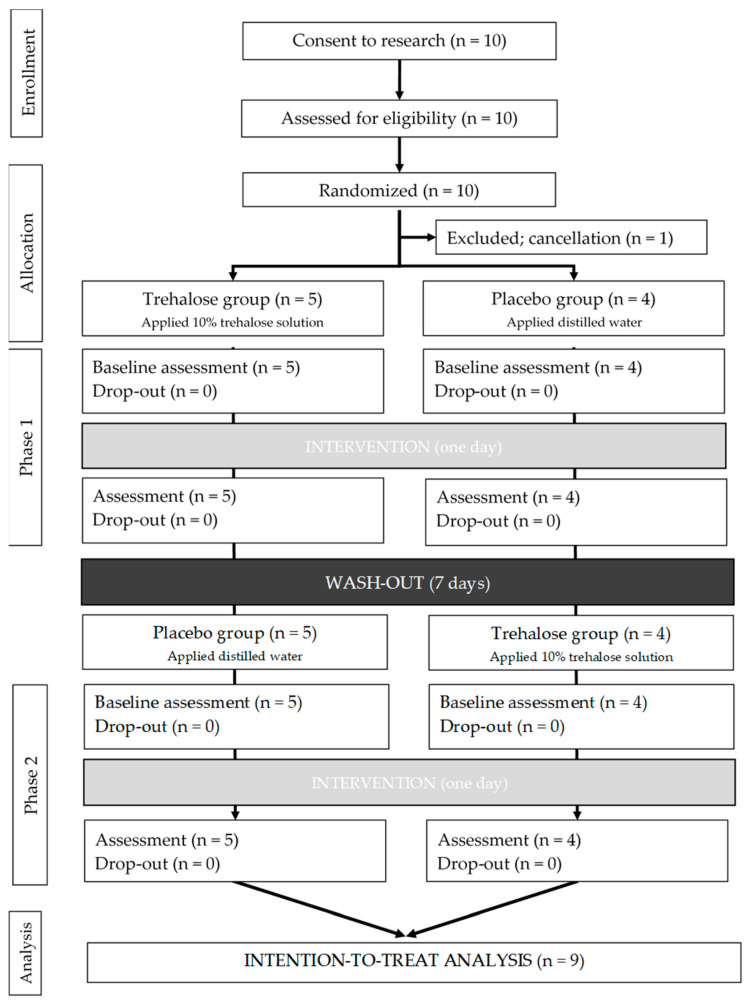
Flow chart.

Figure 2 shows the flow chart for the study. Ten participants were enrolled, and then recruitment was stopped. After the allocation, one participant withdrew. The final ITT analysis was thus performed using data from 9 participants.

### 3.2. Baseline Data

Table 1 shows the baseline data. The participants did not report awareness about halitosis. The organoleptic scores of all of the participants were >1, and they were diagnosed as constituting halitosis. No significant differences in variables were apparent between the trehalose and placebo groups (*p* > 0.05).

### 3.3. Outcomes

Table 2 shows the study outcomes. After the application of 10% trehalose as an oral spray, the organoleptic score was seen to decrease in a time-dependent manner. However, no significant differences were seen between the trehalose and placebo groups. In terms of the secondary outcomes, the oral moisture levels increased immediately after the trehalose spray application, and a significant difference in the amount of change from the baseline was seen between the trehalose and placebo groups (*p* = 0.047). Five participants reported an alleviation of xerostomia immediately after the trehalose spray application. No significant differences were seen in the other variables (*p* > 0.05).

### 3.4. Harms

No adverse events or unintended effects were encountered during the trial. All of the participants completed all of the procedures without any difficulties.

## 4. Discussion

To the best of our knowledge, this represents the first study to examine the effect of trehalose on halitosis in a prospective, double-blinded, placebo-controlled, cross-over study. After the application of 10% trehalose as an oral spray, the organoleptic scores decreased in a time-dependent manner. However, no significant differences were seen between the trehalose and placebo groups. On the other hand, among the secondary outcomes, the oral moisture levels increased immediately after the trehalose spray application, and significant differences in the amount of change from the baseline were seen between the trehalose and placebo groups.

One spray of 10% trehalose reduced halitosis, but the trehalose showed no independent effect on halitosis because the placebo (distilled water) also reduced halitosis. These findings suggest that a one-time application is insufficient to reduce halitosis beyond the effect of a placebo. The bioprotective properties of trehalose have led to the wide use of this saccharide in biotechnology, pharmaceutical, food, and cosmetic applications, as well as in medicine [29]. Further studies are required to determine whether improvements can be achieved in the application time, concentration, and/or method of application.

One spray of 10% trehalose immediately improved the oral moisture levels, and a significant difference was identified between the trehalose and placebo groups. Trehalose has the effects of moisturizing and inhibiting oral dryness [16]. Our findings supported the previous results [16]. Further, five participants reported an alleviation of xerostomia. One application of the 10% trehalose spray thus achieved at least transient effects on oral dryness or xerostomia.

No effects on the VSC concentrations were seen from a one-time application of 10% trehalose by oral spray in this study. Trehalose has been used in the treatment of dry eye disease as a method of stabilizing the lipid layer and reducing the osmolarity of tear film, helping to prevent evaporation [29]. Our expectation was that trehalose would prevent the volatilization of VSCs, but no such effect was identified in this study. Natural trehalose has not been reported to have direct antimicrobial effects, although trehalose is present in a wide variety of organisms, including bacteria [30]. Some trehalose analogs bearing modifications are known to exert direct effects on bacteria [31,32]. Because VSCs are metabolic products of oral bacteria [33], new bioactive trehalose-based products might be anticipated to affect oral bacteria and the production of VSCs.

The strengths of this study included its prospective, double-blinded, placebo-controlled, cross-over design. In addition, an independent dentist reviewed the safety data throughout the trial, and no adverse effects were encountered during this trial. Third, no drop-outs occurred after the baseline examination, and per protocol analyses were thus unnecessary. Finally, the trial demonstrated a low risk of biases (arising from the randomization process, due to deviations from the intended interventions, due to missing outcome data, and in the measurement of the outcome) as per the Cochrane risk bias tool [34].

This study did have some limitations that should be kept in mind when interpreting the results. First, the mean age (±SD) of the participants in the present study was 23.6 years (±1.1), the participants represented a narrow range of the population, and different results may be obtained in middle-aged and elderly participants. Second, we only used a single application of oral spray because we sought to determine whether 10% trehalose can be applied in clinical settings to modify halitosis. Other possibilities that should be investigated in future studies include repeated spray applications or the use of a rinse. Third, all of the participants were recruited from Okayama University Hospital. The generalizability of the results may thus be limited. Fourth, there are no data regarding important cofounders, including oral microbiomes. Finally, although a sample size calculation was performed, the sample size was small, with only 9 participants. Large-scale clinical trials are needed to validate the results of this study.

## 5. Conclusions

We could not identify any positive effects of a one-time application of 10% trehalose by oral spray on halitosis in our prospective, double-blinded, placebo-controlled, cross-over study. However, the trehalose application immediately improved the oral moisture level, and a significant difference was seen between the trehalose and placebo groups.

## Figures and Tables

**Table 1:** Participant characteristics at baseline (n = 9).

Variable	Total	Trehalose	Placebo
Age (years)	23.6 ± 1.1	-	-
Male/Female	8 (88.9)/1 (11.1)	-	-
Systemic diseases	0 (0.0)	-	-
Organoleptic score	2.6 ± 0.8	2.7 ± 0.7	2.6 ± 0.8
Organoleptic score > 1	9 (100.0)		
Volatile sulfur compounds (ppb)			
Hydrogen sulfide	66.6 ± 72.5	56.2 ±89.9	77.0 ± 53.5
Methyl mercaptan	38.5 ± 37.6	46.8 ± 51.5	30.2 ± 14.1
Dimethyl sulfide	6.0 ± 9.7	4.8 ± 4.0	7.2 ± 13.4
Oral moisture level	30.0 ± 1.3	29.9 ± 1.6	30.1 ± 1.0

Values are given as mean ± standard deviation or number (percentage).

**Table 2:** Differences in primary and secondary outcomes between trehalose and placebo groups (n = 9).

Variable	Trehalose	Placebo	p *	Trehalose; Change from Baseline	Placebo; Change from Baseline	p *
Immediately after						
Organoleptic score	1.8 ± 0.8	1.8 ± 0.7 ^1^	0.924	−0.9 ± 0.8	−0.8 ± 0.4	0.799
Volatile sulfur compounds (ppb)						
Hydrogen sulfide	48.3 ± 43.3	32.3 ± 31.9	0.424	−7.9 ± 71.2	−44.7 ± 67.7	0.233
Methyl mercaptan	38.9 ± 38.9	29.1 ± 37.3	0.231	−7.9 ± 32.1	−1.1 ± 36.5	0.895
Dimethyl sulfide	8.1 ± 7.1	12.6 ± 28.2	0.248	3.3 ± 8.0	5.3 ± 18.0	0.756
Oral moisture level	30.3 ± 1.3	29.8 ± 0.8	0.215	0.4 ± 0.9	−0.3 ± 0.7	0.047
At 30 min						
Organoleptic score	1.9 ± 0.8	1.7 ± 0.7	0.534	−0.8 ± 0.7	−0.9 ± 0.9	0.591
Volatile sulfur compounds (ppb)						
Hydrogen sulfide	37.9 ± 51.6	64.9 ± 69.9	0.397	−18.3 ±57.1	−12.1 ± 81.5	0.895
Methyl mercaptan	56.3 ± 65.1	25.6 ± 10.8	0.479	9.6 ± 58.7	−4.7 ± 17.0	0.479
Dimethyl sulfide	3.1 ± 5.5	8.9 ± 14.3	0.609	−1.7 ± 6.1	1.7 ± 5.1	0.168
Oral moisture level	29.5 ± 1.3	29.5 ± 1.3	0.895	−0.4 ± 0.8	−0.4 ± 0.9	0.894
At 60 min						
Organoleptic score	2.0 ± 0.7	1.9 ± 0.8	0.737	−0.7 ± 0.7	−0.7 ± 1.0	0.962
Volatile sulfur compounds (ppb)						
Hydrogen sulfide	49.2 ± 47.5	72.2 ± 49.3	0.232	−7.0 ± 60.4	−4.8 ± 77.2	0.860
Methyl mercaptan	54.6 ± 64.9	27.8 ± 14.5	0.691	7.8 ± 51.5	−2.4 ± 18.6	0.757
Dimethyl sulfide	3.8 ± 4.8	4.9 ± 9.5	0.616	−1.0 ± 6.1	−2.3 ± 4.4	0.857
Oral moisture level	29.5 ± 1.8	29.4 ± 1.6	0.596	−0.4 ± 1.5	−1.0 ± 1.0	0.233
At 90 min						
Organoleptic score	2.2 ± 0.8	1.9 ± 0.8	0.373	−0.4 ± 0.8	−0.7 ± 1.0	0.609
Volatile sulfur compounds (ppb)						
Hydrogen sulfide	71.8 ± 45.8	101.6 ± 62.9	0.353	15.6 ± 89.7	24.6 ± 71.1	0.860
Methyl mercaptan	38.7 ± 43.6	35.4 ± 24.9	0.479	−8.1 ± 28.0	5.2 ± 17.3	0.479
Dimethyl sulfide	8.4 ± 11.7	8.9 ± 11.5	0.824	3.7 ± 13.0	1.7 ± 4.1	0.535
Oral moisture level	29.0 ± 1.8	29.9 ± 2.0	0.353	−0.9 ± 1.3	−0.4 ± 1.1	0.426

Values are given as mean ± standard deviation or number (percentage); * Mann–Whitney *U* test.

## Data Availability

The data supporting the findings of this study are available from the corresponding author (D.E.) on request.

## Abbreviations

The following abbreviations are used in this manuscript:

VSCs	volatile sulfur compounds
ITT	intention-to-treat
